# Supplementary material for: Radiocesium transfer rates among pigs fed haylage contaminated with low levels of cesium at two differentiation stages
Source: PLoS One. 2020 Sep 11;15(9):e0237977. doi: 10.1371/journal.pone.0237977 (PMC7485866; doi:10.1371/journal.pone.0237977)
Supplement: Attachment — Submitted filename: Response to Reviewers.docx [file pone.0237977.s001.docx]

I would like to thank the reviewers.

I am prepared the manuscript according to the PLOS ONE's style requirements and I believe that revised manuscript could be meet your requirement for publishing in journal PLOS ONE. The details answer to reviewer’s comments is as below.

Reviewer #1:

1. The value and significance of this study should be proposed in the last sentence of the Abstract.

Answer: I will answer this question with fourth question together.

2. The first three paragraphs of Introduction needed to be properly integrated.

Answer: The first three paragraphs of Introduction already integrated.

3. Although the authors described a series of indicators tested in this study in the last paragraph of Introduction, there was still a need to describe the meaning behind these indicators.

Answer: All of the indicators are described in the Materials and Methods section.

4. Why did the author select only four adult pigs? In addition, in the absence of a control group, how could the author ensure that the detected indicators are statistically significant?

**Answer(1&4):** The cesium, especially cesium-137 and cesium-134, a fission product, are extracted from waste produced by nuclear reactors. Thus the pigs could not intake cesium from the nature. The second, present study and previous study (Li et al., 2019) was confirmed when the pigs fed with none contaminated food there was no organs radiocesium contamination was detected. Third, the purpose of present study is to know the radiocesium transfer rates among pigs when fed haylage contaminated with low levels of cesium at two differentiation stages. Fourth, the nuclear power plant disaster and release of large amounts of radioactive material is the very few and moment case in the world history. The present study, the purpose is to know the radiocesium transfer rates in organ tissues of pigs when fed with haylage contaminated with low levels of Cs for 1 month at different growth stages. And the Animal Care and Use Committee of the Graduate School of Agricultural and Life Sciences at the University of Tokyo, ask every project to reduce the number of animals scarifying as could as possible. Meanwhile the hematological and biochemical indices of the pig already have standard value for the pig clinical check. The present study just check the every pigs’ health condition not need to compare with control group. Thus present study absence of control group and significance value and statistically significance.

5. Why the authors chose 0.7 kg of contaminated haylage to feed the pigs.

Answer: Before the examination, the juvenile pig fed with 1:1 haylage and concentrate mixture and confirmed that average feed intake was 1.4kg. This is the reason why present study feed pigs with 0.7kg contaminated haylage.

6. Why was the current point of time chosen to collect the blood sample?

Answer: Because there is no data how cesium concentration affect animal health. The present study referenced our previous study (published) and Animal Care and Use Committee of the Graduate School of Agricultural and Life Sciences at the University of Tokyo, ask every project to reduce the number of blooding times from animals as could as possible. The present study blood time is the minimum times that decided by the authors’ knowledge.

7. The first paragraph of the Discussion seemed more appropriate in the Materials and Methods section.

Answer: already done.

8. A general conclusion should be added to the last paragraph of the Discussion.

Answer: The general conclusion has added to the Discussion section.

9. All Tables should use a three-line table and, in addition, the number of biological duplicates should be indicated in the table notes.

Answer: already done.

10. The captions and order of Figure 1 and 2 were incorrect and needed to be corrected.

Answer: already corrected.

Reviewer #2: There is no explanation for many abbreviations in the table and figures.

Answer: All of the abbreviations in the table and figures please reference to the material and methods section.

Figure 1- what does stop treatment in the legend

Answer: the legend of the Figure 1 is changed to [stopped fed contaminated haylage]

Table 1 & 2- what are units for weight

Answer: added the unit of the weight in the tables.

Table 3 & 4- what are (T) and (C) in column 2.

Answer: added the legends of the (T) and (C) to the tables.

Table 5- what is SD in column 1

Answer: I am so sorry. It is a mistake. The SD should be SE (standard error) and it has corrected.
